# Draft Genome Sequence of Vibrio harveyi Strain GAN1709, Isolated from Diseased Greater Amberjack (Seriola dumerili) Farmed in Japan

**DOI:** 10.1128/genomeA.00611-18

**Published:** 2018-06-28

**Authors:** Shinya Monno, Hidehiro Sugiura, Hazuki Yamashita, Yusuke Kato, Kazuyuki Morimitu, Masayuki Imajoh

**Affiliations:** a Laboratory of Fish Disease, Faculty of Agriculture, Kochi University, Nankoku, Kochi, Japan; b Department of Bioresource Production Science, The United Graduate School of Agricultural Sciences, Ehime University, Matsuyama, Ehime, Japan; c Kanei Suisan, Susaki, Kochi, Japan

## Abstract

Vibrio harveyi strain GAN1709 was isolated from a diseased greater amberjack farmed in Nomi Bay, Japan. Here, we report the draft genome sequence of this strain, which comprises 6,265,473 bp, with a G+C content of 44.8%.

## GENOME ANNOUNCEMENT

Vibrio harveyi, which is a luminous Gram-negative halophilic bacterium, causes vibriosis in commercially farmed marine vertebrate and invertebrate species (1). The emergence of vibriosis in greater amberjack (Seriola dumerili) farmed in Japan became a serious problem in approximately 2010 (2, 3). This disease occurs mainly in summer, during periods when water temperatures are high (2) and diseased fish present symptoms, such as exfoliation of the head epithelium, ragged caudal fins, accumulated ascites, and ophthalmitis (3).

Nomi Bay, which has an area of approximately 4 km^2^ and an average depth of approximately 18 m, is an area used for fish mariculture in Kochi Prefecture, Shikoku Island, Japan (4). A dead greater amberjack with a weight of 510 g and symptoms that included a ragged caudal fin and severe ophthalmitis was collected in the bay on 6 September 2017. A Vibrio isolate was collected from the eye lesions of the dead fish and cultured on thiosulfate-citrate-bile salts-sucrose (TCBS) agar. The isolate was identified as Vibrio harveyi based on the results of species-specific PCR assays of DNA topoisomerase I and rod-shaped-determining-protein genes that were reported in a previous study (3). Here, we report the draft genome sequence of this isolate (strain GAN1709).

A single colony of strain GAN1709 grown on TCBS agar was selected and transferred to 10 ml of fresh brain heart infusion broth with an additional 1.5% sodium chloride and incubated overnight at 27°C. Genomic DNA was extracted using a Qiagen Genomic-tip 500/G kit and a genomic DNA buffer set. Genome sequencing was performed on an Illumina MiniSeq platform (Illumina K.K., Tokyo, Japan), which generated 6,064,921 reads. The sequencing reads were assembled using CLC Genomics Workbench 10.1.1 (Qiagen, Tokyo, Japan). The assembly of strain GAN1709 consisted of 256 contigs totaling 6,265,473 bp, with a G+C content of 44.8%. The draft genome sequence was annotated using Microbial Genome Annotation Pipeline version 2.23 (http://www.migap.org/), which yielded 5,824 protein-coding sequences (CDS), 73 tRNA genes, and 4 rRNA operons.

V. harveyi-associated vibriosis has been reported in many aquaculture species in China and is therefore considered a major disease in the mariculture industry (5). Numerous greater amberjack juveniles are imported every year from southern China to Nomi Bay for farming purposes. The draft genome sequence of V. harveyi strain GAN1709 reported here will be useful for examining whether the imported juveniles are a source of V. harveyi in Nomi Bay.

### Accession number(s).

This whole-genome shotgun project has been deposited in GenBank under the accession number BGNF00000000. The version described in this paper is the first version, BGNF01000000.
